# Changes in tissues and organs through PMCTA carrier substances

**DOI:** 10.1007/s00414-024-03350-9

**Published:** 2024-10-24

**Authors:** G. M. Bruch, N. H. C. Feldmann, F. T. Fischer, T. Fracasso, S. Grabherr, P. Genet

**Affiliations:** 1 Institute of Legal Medicine, Ludwig-Maximillians-University Munich, Nussbaumstr 26, D– 80336 Munich, Germany; 2 University of Geneva, Rue du Général-Dufour 24, CH - 1211 Geneva, Switzerland; 3 University Center of Legal Medicine, University Hospitals of Geneva, Rue Michel-Servet 1, CH – 1211 Geneva, Switzerland; 4 University Center of Legal Medicine, University Hospital of Vaud, Chemin de La Vulliette 4, CH - 1000 Lausanne, Switzerland

**Keywords:** PMCTA, Carrier substance, PEG, Paraffin oil, Polyethylenglycole

## Abstract

To date, lipophilic contrast agents mixed with oil, usually paraffin oil, are the most commonly used contrast agents in post-mortem computed tomography angiography (PMCTA). Iodine-based hydrophilic contrast media in combination with a water-soluble carrier, e.g. polyethylene glycol (PEG), are also common. However, their influence on different tissues and organs is poorly understood. In order to analyse the changes in the cadavers caused by the different carrier substances, we evaluated the effects of PEG 200 and oil on the different tissues and organs. Therefore, during a forensic autopsy, liquid femoral vein blood and samples of different organs and vessels were taken and preserved at room temperature in the two liquids mentioned. The condition of the samples was documented during the autopsy and 24, 48 and 72 h after preservation. Microscopic examination took place after 72 h. After 24 h, the samples placed in PEG 200 already showed a clear solidification of almost all structures. Crumbly blood agglomerates had formed in the previously liquid blood. In contrast, the samples stored in oil showed signs of classic cadaveric decomposition after 24 h, which increased with time. The microscopic and immunohistochemical evaluation of the samples stored in PEG showed a good diagnostic quality. The analysis of tissues stored in oil was much more difficult due to putrefaction. PEG and oil show significantly different effects on human tissues, mainly conservation and dehydration are affected. It is crucial to be aware of these differences in order to choose the most appropriate PMCTA method for each forensic case.

## Introduction

Decisive developments in forensics, especially in forensic imaging, took place at the beginning of the 2000s, when the Virtopsy Group in Switzerland further developed methods of postmortem imaging, including postmortem angiography [1, 2].

Since then, routine work regarding postmortem imaging in forensic medicine has steadily evolved [1, 3–6]. Post-mortem computed tomography (PMCT) with the injection of a contrast medium allows detailed evaluation of the vessels and is therefore useful for detecting vascular injuries or abnormalities and bleeding. In forensic medicine, this enables thorough analysis of deaths after surgical interventions, ballistic and sharp trauma, as well as for cases with vascular malformations and aneurysms. Furthermore, Post-mortem Angiography (PMA) ensures the evaluation and illustration of coronary arteries in detail in cases of sudden cardiac death [7, 8].

Different substances were tested to perform PMCTA (Post-mortem Computed Tomography Angiography) [4, 9–13]. The Virtopsy Group tested meglumine-ioxithalamate [1] and lipophil diesel oil in combination with a clinical contrast medium [13] and polyethylene glycol mixed with a clinical contrast medium [10]. Later, the research group of Grabherr et al. introduced a new mixture consisting in paraffin oil mixed with the newly invented oil-based contrast medium Angiofil® [4].

Currently, mainly two different carrier substances, oil, e.g., paraffin and vegetable oil, and polyethylene glycol 200 (PEG) are frequently used in forensic medicine to perform whole-body PMCTA [3, 4, 10, 14, 15]. The contrast media used are mainly Angiofil® for the mixture with oil and Accupaque®, a clinical contrast medium mixed with PEG.

Studies have shown the advantages and disadvantages of lipophilic contrast media, e.g., Angiofil® mixed with paraffin oil [3, 4, 7, 16]. Mainly due to its advantages, such as intravascular retention of the lipophilic contrast medium in mixture with paraffin oil and its easy handling as well as its low viscosity, this mixture is regularly used for PMCTA. However, one of the main disadvantages of oil mixed with oil-based Angiofil® is its critical applicability in is cases where a (fatal) fat embolism needs to be investigated. This can be in cases of trauma with a need to investigate vital signs or in cases of death after surgery, where fatty embolism may have played a role. Indeed, as the oily contrast medium produces fatty embolism itself, it results in a false-positive histological result and should therefore not be used for investigating such cases, unless samples of pulmonary tissue have been collected previously [17]. However, the procedure of sampling may cause artefacts that are difficult to interpret and therefor are better to be avoided.

To the best of our knowledge however, PMCTA performed with polyethylenglycol mixture has not been described in detail until today [3, 10, 14]. It is known that PEG-based mixtures have hygroscopic abilities. They can bind water and lead to clumping [18]. To date, no one has compared the effects of this carrier substance with those of the oil used for lipophilic contrast media mixtures. However, it is important to know the differences between the various carrier substances in order to choose the most suitable PMA method, depending on the forensic case treated.

The aim of our study was therefore to describe and compare the influence of the carrier substances “PEG” and “oil” on different organs, tissues and vessels in an in vitro experiment.

## Materials and methods

Ethical aspects: Principal consent of the local ethics committee was obtained, as the committee stated, that “examinations of body materials and evidence from cadavers taken and examined on behalf of public prosecutors or investigating authorities [for scientific purpose] there is no obligation to seek advice of the Ethics Committee in case of scientific publication of anonymized results” (No. 22–0572-KB).

During a prosecutor-ordered autopsy (male, 48 years, 2,5d pm-interval, cause of death: mechanical asphyxia due to hanging), where no PMCTA was performed, tissue, organ and vessel samples were taken at the Institute of Legal Medicine Munich. Changes in the organs caused by the carrier substance polyethylenglycol 200 g/mol (PEG 200; Merck KGaA, Darmstadt, Germany) with a viscosity of 60–67 mPas (20°) and oil (rapeseed oil; P. Brändle GmbH, Empfingen, Germany) with a viscosity of 60 mPas (20°) were documented. Neither the skin nor the inner organs of the corpse showed any signs of putrefaction during the external examination or the autopsy.

Liquid blood from the inferior vena cava as well as samples (each appx. 3 cm in diameter) of the following organs, tissues and vessels were taken:

Brain, lung, liver, spleen, kidney and fatty tissue, heart muscle, tissue of the stomach wall and of the vessel wall of the inferior vena cava, the main carotid artery and the abdominal aorta were removed and stored at room temperature (21 °C) in two liquid-filled jars, one filled with PEG 200 and one filled with rapeseed oil. The liquid vein blood was mixed at a ratio of approximately 1/3 blood to 2/3 carrier substance.

During the autopsy, all the samples had tissue-specific haptic and tissue-specific optic. The lung samples were also rich in blood. The arterial walls showed partial signs of discrete atheromathosis.

The condition of the samples was assessed during autopsy and at 24 h, 48 h and 72 h after preservation. At the three different time points, the color and consistency of the organs and tissues were described, and all samples were documented photographically.

### Histological analysis

After 72 h in the liquid, microscopic analysis followed. All the samples underwent hematoxylin and eosin (HE) staining. After that, individual organ specific immunohistology staining followed, depending on the quality of the HE staining.

## Results

**Fig. 1:**
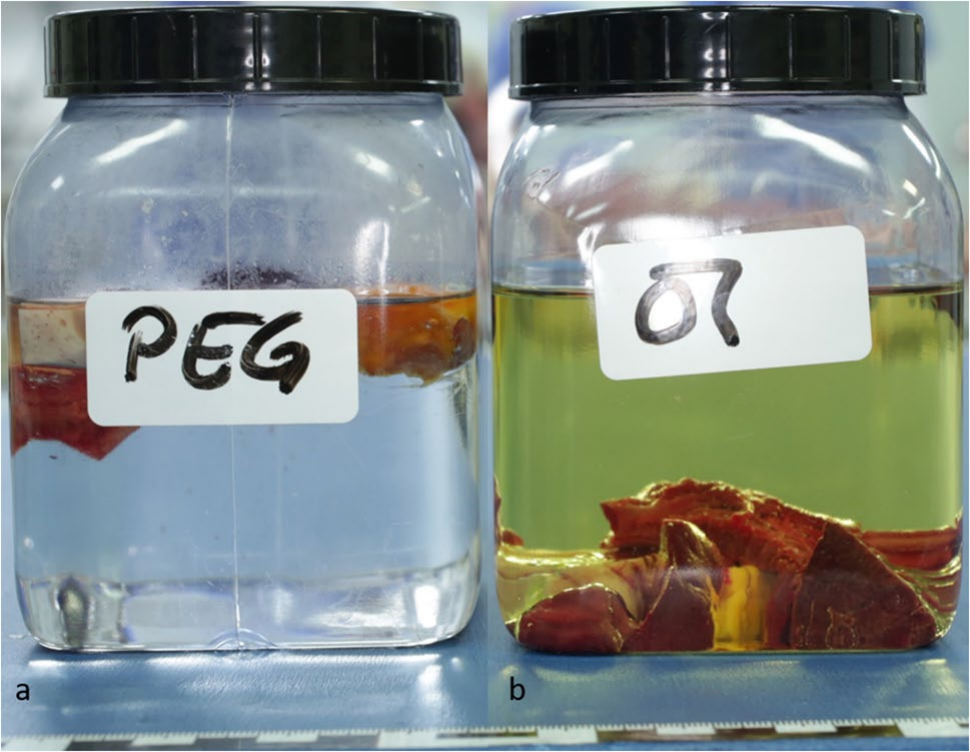
Tissues after autopsy **a**) stored in PEG, swimming on top of the fluid **b**) stored in Oil, sinking to the bottom of the jar

Immediately after placing the different tissues, organs and vessel walls in the corresponding liquids, it was noticeable that the samples in the PEG-filled jar floated to the top (Fig. 1a). The samples that were placed in oil, on the other hand, sank to the bottom of the jar (Fig. 1b).

### Samples during autopsy

#### Blood

The blood was liquid without emboli or postmortem clots.

#### Brain

During autopsy, the brain tissue was stiff and retained its shape (Fig. 3a).

#### Heart, lung, liver and kidney

The heart muscle (Fig. 4a) and kidney and liver samples showed soft tissue-specific consistency during autopsy. The kidney were additional rich in blood. The lung tissue was moderately rich in blood and water during the autopsy.

#### Spleen and stomach wall

The spleen was paled and softened at autopsy. The stomach wall was folded, without any signs of putrefaction.

#### Fat tissue

The fat tissue showed tissue-specific consistency during autopsy (Fig. 5a).

#### Aorta abdominalis, A. carotis and V. cava inferior

The arterial wall of the aorta and the carotid artery (Fig. 6a A. carotis) had a few streaky fat deposits, observed during the autopsy. The venous vessel (V. cava inferior) exhibited a normal appearance.

### Carrier substance: oil

#### Blood

**Fig. 2:**
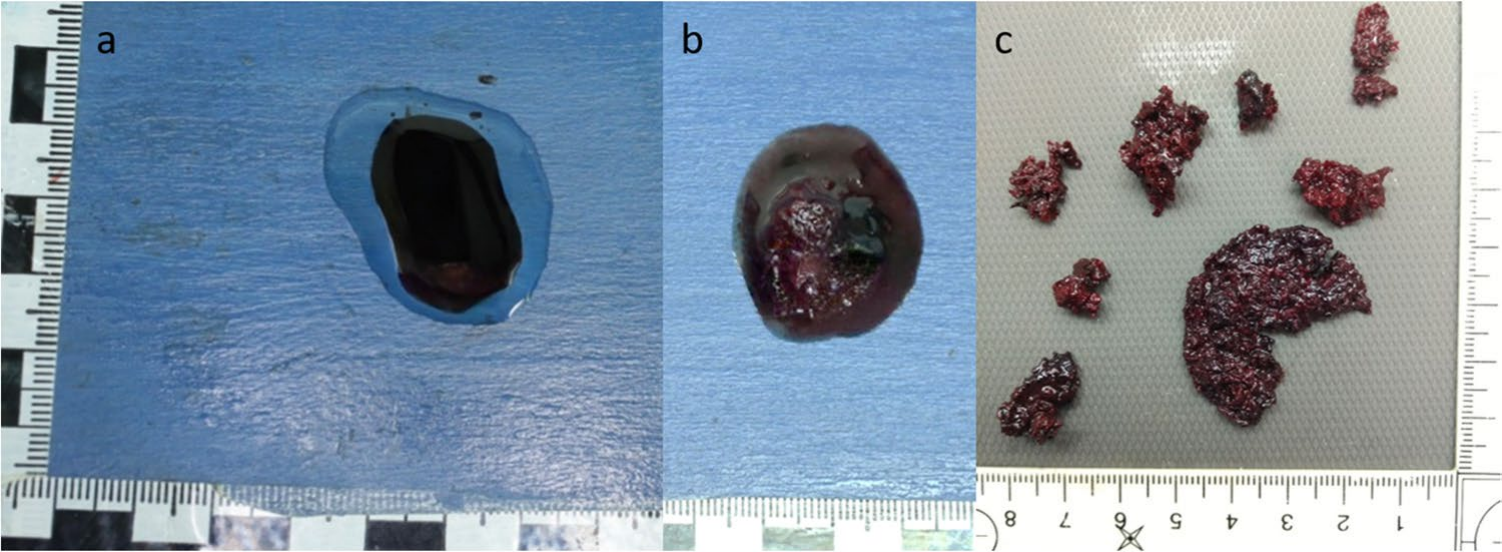
Liquid Blood: **a**) 24 h in oil – **b**) 24 h in PEG – **c**) 72 h in PEG

The liquid blood, which was added to the oily carrier substance during the autopsy, had settled on the bottom of the transparent storage jar after 24 h (Fig. 2a). After shaking the jar, the two liquids mixed again. After 48 and 72 h in oil, the heterogeneous mixture of the substances separated again into two phases. The blood itself remained liquid during the observation period, and no clots appeared. There were no macroscopically visible changes except for the oily appearance of the blood-oil mixture after shaking.

#### Brain

**Fig. 3:**
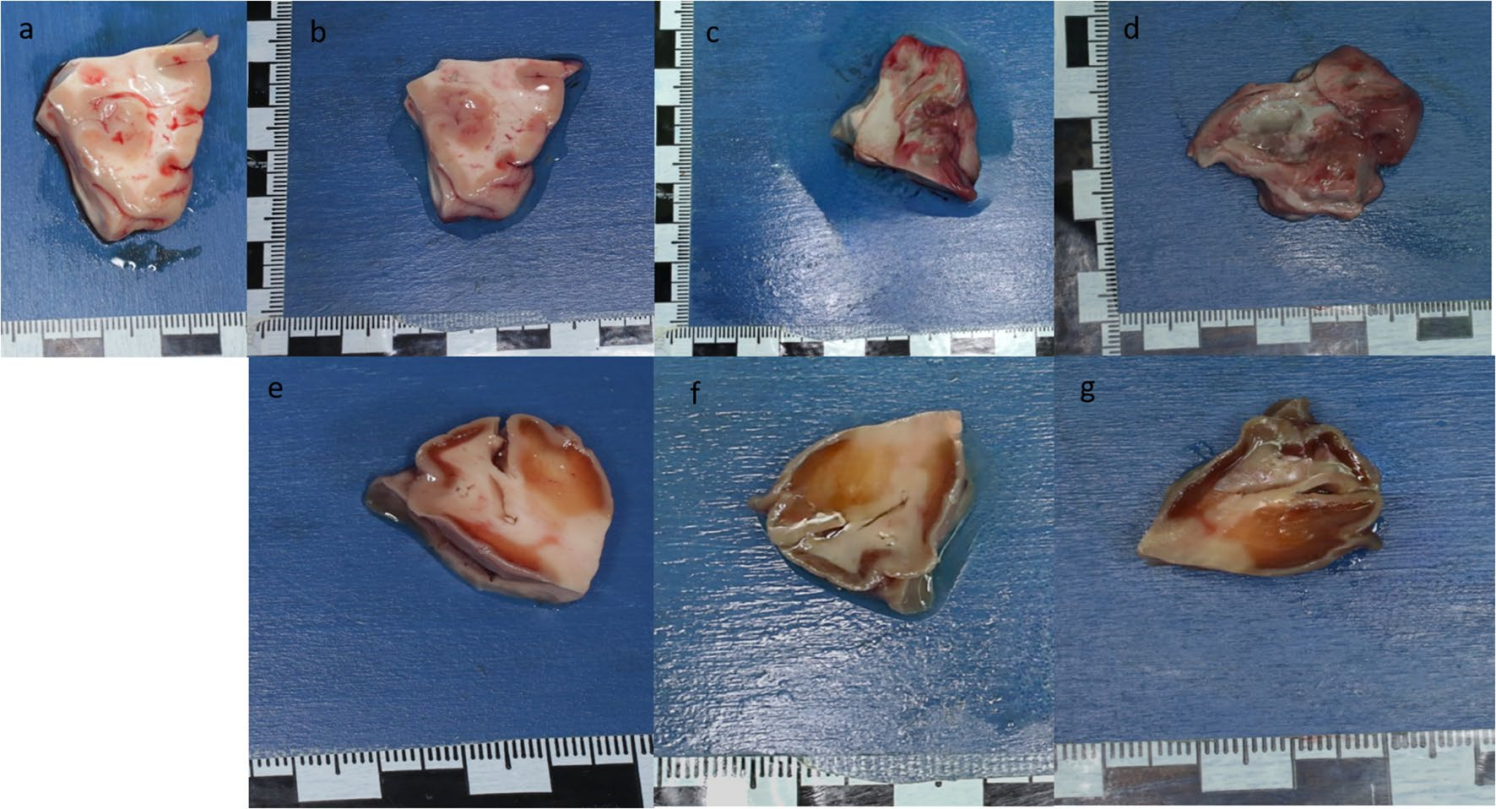
Brain tissue: **a**) during autopsy – **b**) 24 h in oil – **c**) 48 h in oil – **d**) 72 h in oil **e**) 24 h in PEG – **f**) 48 h in PEG – **g**) 72 h in PEG

After 24 h in oil, the white matter started to have a reddish color (Fig. 3b). The gray matter showed no changes. When touching the brain tissue, it seemed softer than it was during autopsy. After 48 h in the presence of the oily carrier substance, the gray matter was red discolored, and the white matter was green‒gray-red, both of which are clear signs of putrefaction (Fig. 3c). After 48 h, the substance could not reach its original form (Fig. 3d). The brain tissue was mushy and red‒green, and clear signs of putrefaction appeared after 72 h.

#### Heart, lung, liver and kidney

**Fig. 4:**
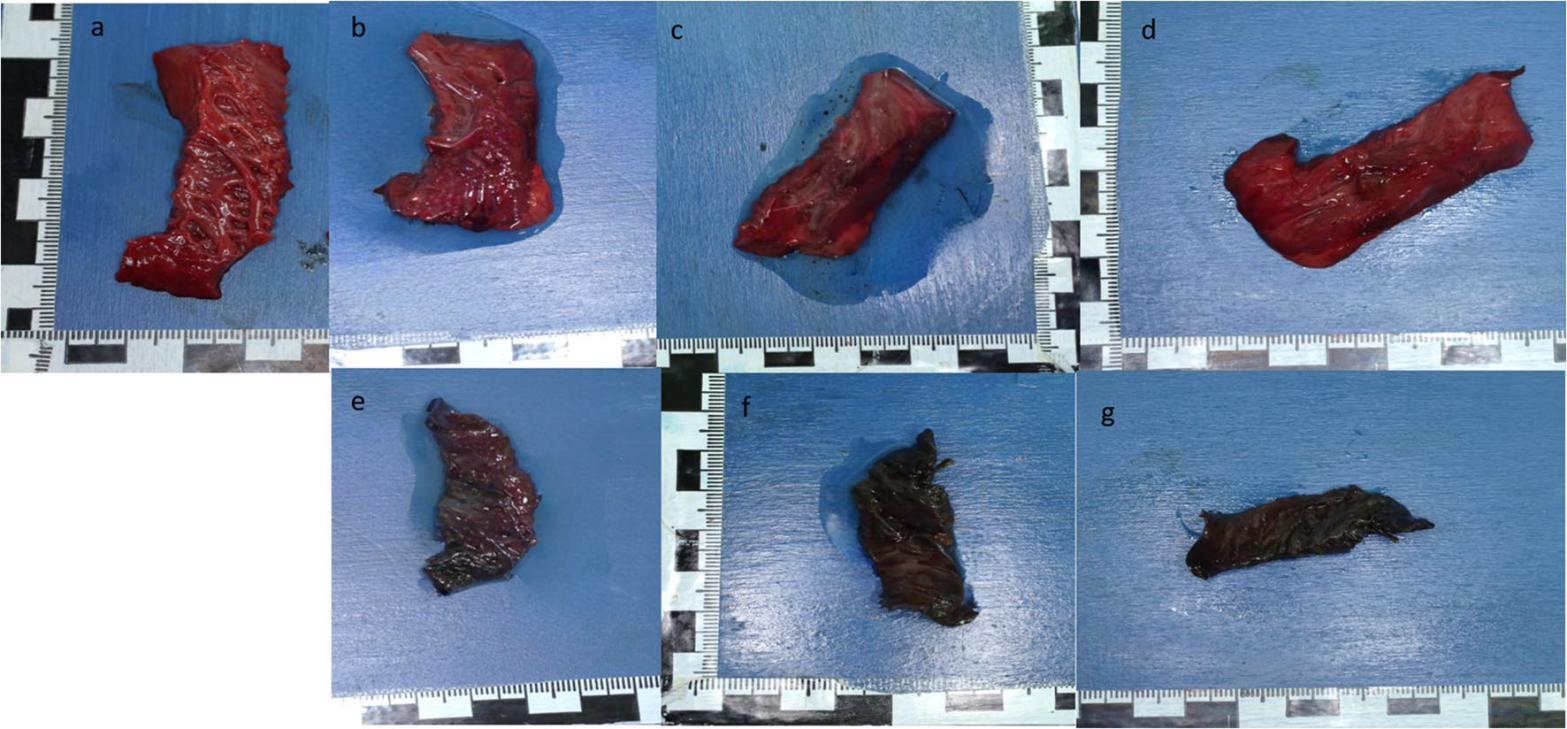
Heart muscle: **a**) during autopsy – **b**) 24 h in oil – **c**) 48 h in oil – **d**) 72 h in oil—**e**) 24 h in PEG – **f**) 48 h in PEG – **g**) 72 h in PEG

A beginning fade was observed in these four tissues after 24 h in oil. The surfaces of these samples were clearly shiny-oily and therefore slippery. Macroscopically, the heart muscle (Fig. 4b) and the lung and liver tissue started to show changes in color. Heart and liver were partly brownish discolored on the edges after 24 h. This discoloration increased after 48 h, and after 72 h, softening was felt, and a clear change in color was observed in these tissues (Fig. 4c & d for the heart tissue). The kidney changed color to a homogeneous brown over time and softened but was still identifiable.

#### Spleen and stomach wall

The white pulp of the spleen was still visible after 24 h in oil. Over time, the tissue became dark brown, softened and was no longer macroscopically identifiable. After 24 h, the stomach wall was softened, reddish discoloured and hardly identifiable. This appearance increased over time.

#### Fat tissue

**Fig. 5:**
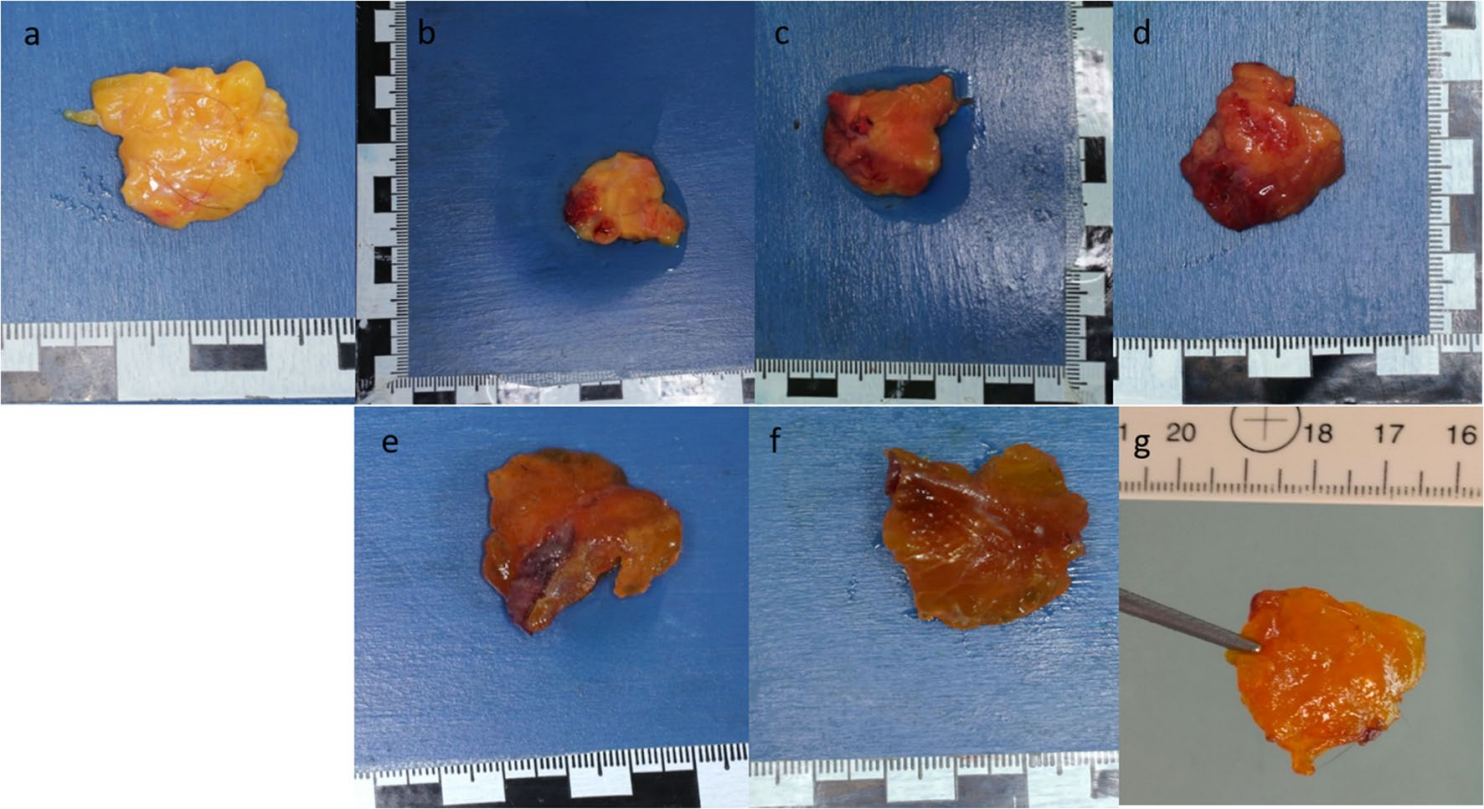
Fat tissue: **a**) during autopsy – **b**) 24 h in oil – **c**) 48 h in oil – **d**) 72 h in oil—**e**) 24 h in PEG – **f**) 48 h in PEG – **g**) 72 h in PEG, held up to the light

After 24 h in oil and after dabbing, the fatty tissue appeared optically dull, and it had lost its original shiny aspect. On the edges, a reddish discoloration was observed (Fig. 5b). The haptic did not change notably. The red discolouration increased after 48 h, and softening was felt (Fig. 5c). After 72 h in oil, the fat tissue changed its color completely to orange‒red with brownish areas (Fig. 5d). The fat tissue was partly decomposed.

#### Aorta abdominalis, A. carotis and V. cava inferior

**Fig. 6:**
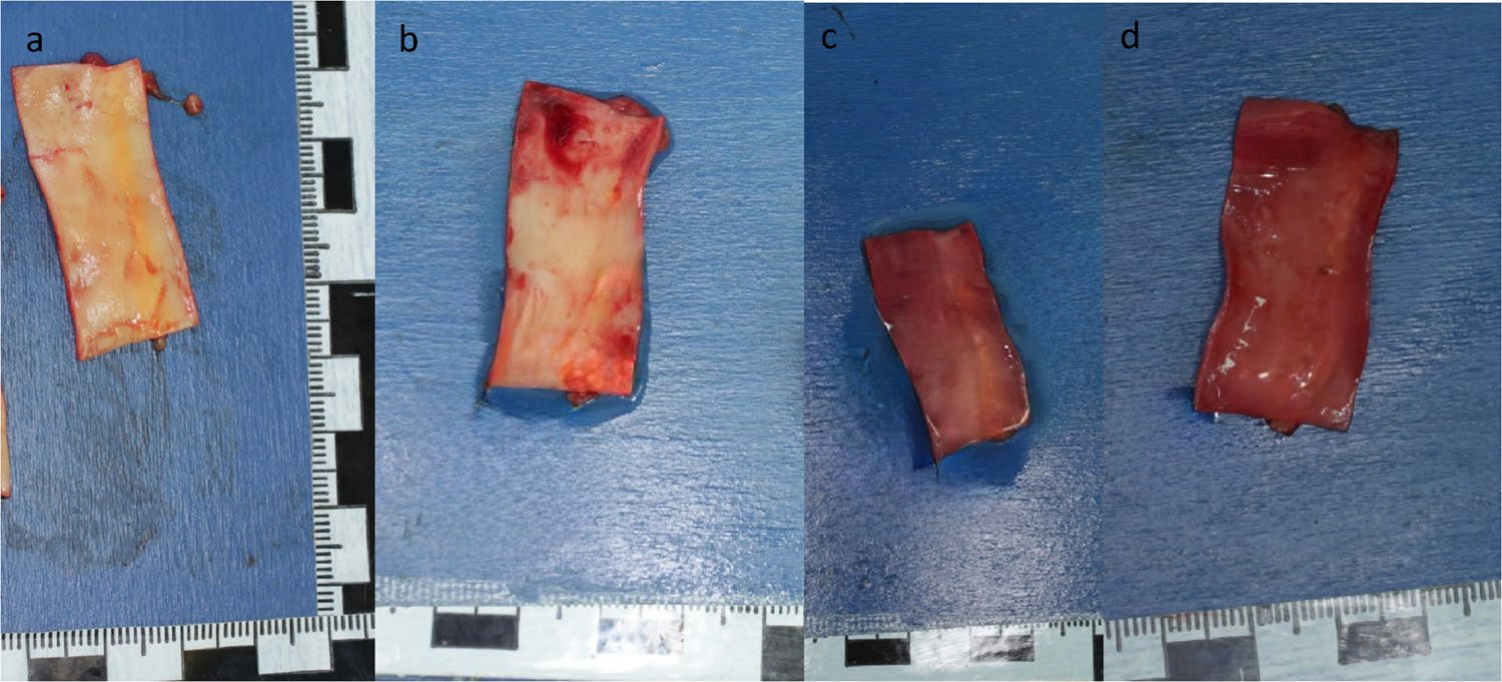
A. carotis: **a**) during autopsy – **b**) 24 h in oil – **c**) 48 h in oil –** d**) 72 h in oil

After 24 h in oil, the arterial wall of the aorta and the carotid artery were already slightly brownish-reddish (Fig. 6b A. carotis), and after 48 h as well as after 72 h, it was clearly discoloured in total as a sign of advanced decay (Fig. 6c and d A. carotis). The fat deposits surrounding the vessel remained visible at all times as soft fat storage and were palpable.

After 24 h, the V. cava inferior a partly dull purple, partly distinct green discoloration of the intima was observed, which was compatible with decay. The coloration darkened after 48 h in oil, and the tissue showed small gas bubbles in the intima. After 72 h in oil, the rotting changes persisted.

### Microscopic examination

Microscopic examination of the samples was performed after 72 h. The organs showed differently advanced stages of decay. Most tissues and organs subjected to hematoxylin–eosin (HE) staining were permeated with gas bubbles. Empty vacuoles were visible in the HE staining of the liver, and it was not possible to distinguish vacuoles resulting from fatty liver or vacuoles created by oily contrast medium; therefore, the diagnosis of fatty liver was difficult. Heart (Fig. 9b) and brain tissue could not be assessed in detail due to the presence of advanced decay. Only the lipofuscin pigment was still recognizable in the heart. In addition, ubiquitous erythrocytes pressed against the vascular wall could be observed in all vascular sections of the organs. There were also circular, optically empty sections in the lungs, which could be compatible with air due to decomposition or could be the result of the oily contrast medium. The venous and arterial vessel walls were still identifiable. The fat tissue adherent to the wall of the vena cava inferior showed signs of an adipocere.

The assessability of the remaining histological sections was clearly limited and was partly impossible due to decomposition. Due to its degree of decomposition, no immunohistological staining was carried out.

### Carrier substance: polyethylene glycol 200

#### Blood

The liquid blood, which remained in PEG 200 for 24 h after the autopsy, had already formed distinct crumbly blood agglomerates after this time (Fig. 2b), comparable to wet sand. The individual sand-like and loosely connected crumbs were firm to the touch. After the blood had remained in polyethyleneglycol for a total of 72 h, a blood cake had formed on the bottom of the jar (Fig. 2c). At the pressure of a fingertip, this blood cake could be broken down into individual, sand-like crumbs, but the small sand-like formations did not dissolve themselves. In addition to the large blood agglomerates described, countless other small agglomerates had formed in the blood-PEG mixture. These compounds appeared to be no longer soluble.

#### Brain

After 24 h, the stiff brain tissue showed sharp edges in the PEG and a brownish discoloration on the border between the white and gray matter (Fig. 3e). After 48 h in PEG, the sample shrunk in size and at the same time hardened upon contact (Fig. 3f). In addition, the brownish discoloration now included wide areas of white matter. After 72 h, the gray matter was highlighted in a more grayish coloration and stood out against the white matter, which had sunken in and showed a brown color and a hardened touch (Fig. 3g).

#### Heart, lung, liver and kidney

After 24 h in PEG, the heart muscle, lung and liver tissue already showed clear hardening of all structures. After 24 h in PEG, the heart muscle also showed a dull brown color (Fig. 4e). After 48 h, but especially after 72 h, macroscopically, the evidence was hardly recognizable as heart muscle (Fig. 4f and g).

During the autopsy, the lung was found to be moderately rich in blood. After 24 h in PEG, the tissue was palpated rubber-like. In addition, on one side, there was a dark purple zone but still shiny arching with the size of a small finger, while the edge of this zone clearly appeared pale in color, with a medium red color and a dull surface. We assume that the PEG in the darker zone could not cause any change on the pulmonary tissue surface due to the contact of another tissue at this location during storage. After 72 h in PEG, the entire sample was dark red in color and had clearly hardened upon palpation.

The asserted liver had already lost substance after 24 h in PEG. The outer edges of the specimen were clearly visible, while the intermediate parts had sunk in. The liver showed a dark brown colouration and clearly hardened upon palpation. After 48 and 72 h, the degree of hardening increased.

Over time, the kidney showed not only a hardened structure but also sharp and prominent edges with a reddish to brownish discoloration.

#### Spleen and stomach wall

After 24 h in PEG, the spleen showed a clear protrusion of the white pulp, and even after 48 h and 72 h in PEG, it was still visible. The structure hardened over time but was always identifiable. In contrast, the stomach wall became brownish and discoloured after 24 h. After 48 h and 72 h in PEG, the tissue hardened and was hardly macroscopically identifiable.

#### Fat tissue

The fatty tissue, which at the time of the autopsy was shining in the familiar yellow hue, was orange-brown after 24 h in polyethylene glycol and solidified but not completely hardened at palpation (Fig. 5e). After 48 h and 72 h in PEG, this impression increased, and the fatty tissue hardened and showed a shiny orange‒brown discoloration (Fig. 5f). In contrast, the fatty tissue was amber-colored (Fig. 5g).

#### Aorta abdominalis, A. carotis and V. cava inferior

**Fig. 7:**
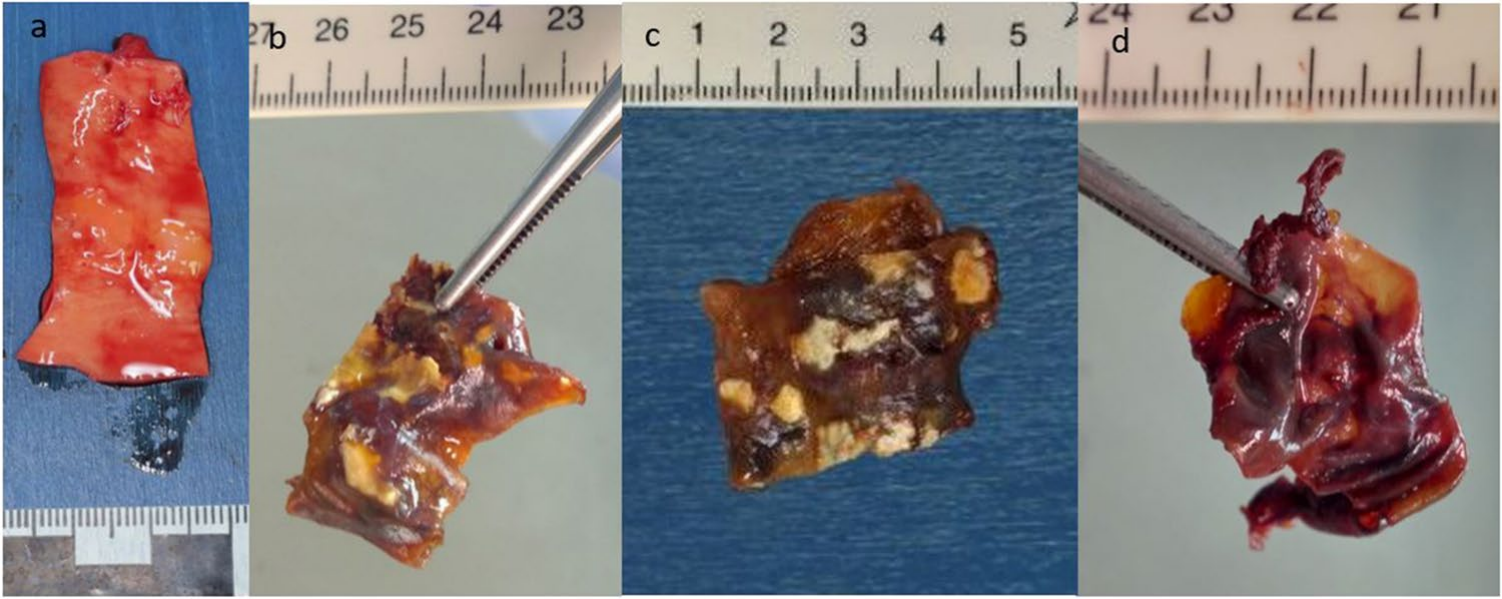
Aorta abdominalis: **a**) during autopsy – **b**) 24 h in PEG, held up to the light – **c**) 48 h in PEG – **d**) 72 h in PEG, held up to the light, backside of the arterie

The Aorta abdominalis (Fig. 7a) and the A. carotis both had fatty strikes. Both changed color to brown‒red with clear visibility of the fat deposits after 24 h in PEG (Fig. 7b Aorta abdominalis). When held against the light, the wall of the vessel shimmered through a parchment. The asserted abdominal aorta appeared to be translucent, and after 48 h and 72 h in PEG, light yellow fatty wall inclusions were clearly visible on the now-brownish, tanned vessel wall (Fig. 7c & d Aorta abdominalis).

**Fig. 8:**
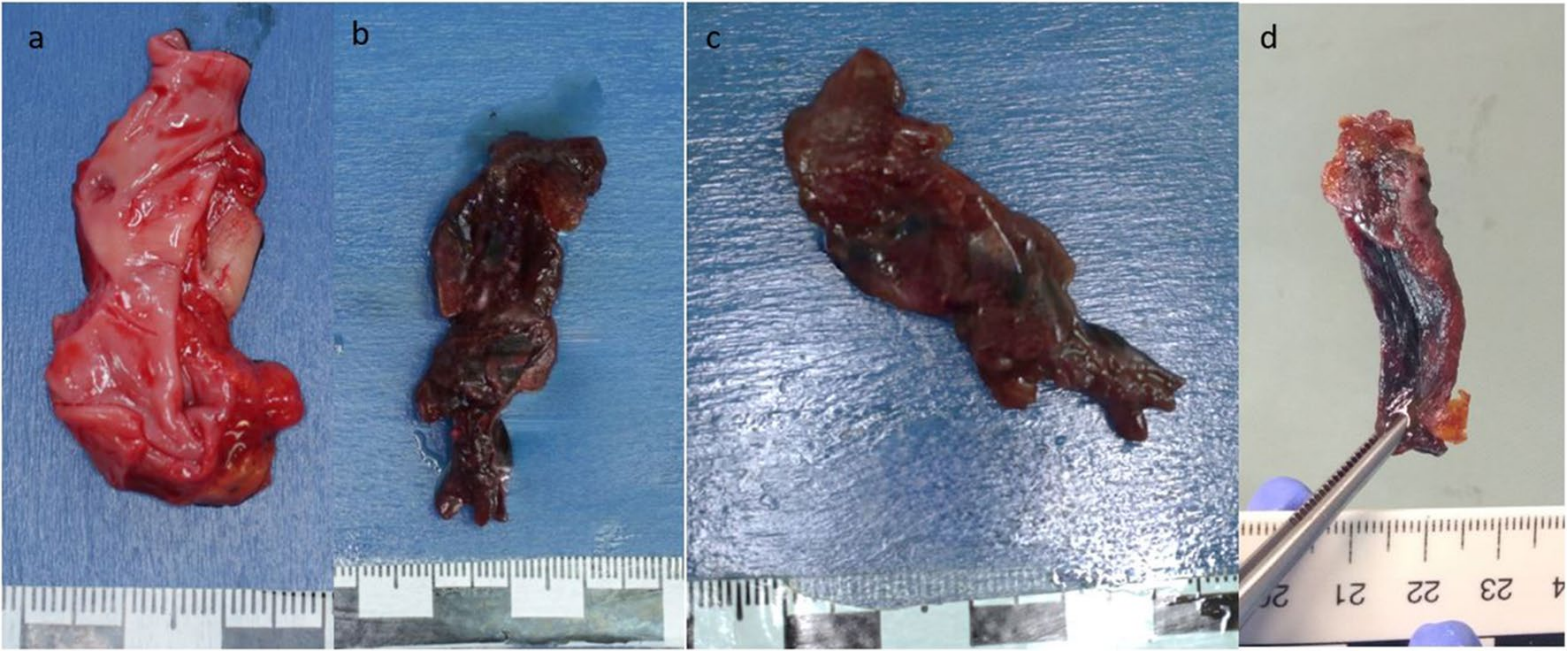
Vena cava inferior: **a**) during autopsy – **b**) 24 h in PEG – **c**) 48 h in PEG – **d**) 72 h in PEG, held up to the light

The wall of the vena cava inferior (Fig. 8a) was already palpated and hardened after 24 h (Fig. 8b), and the hardening increased after 48 h and 72 h in PEG (Fig. 8c and d). The venous vessel did not break apart when the tissue was bent. The vessel wall started to crumble after 24 h in PEG, which increased over time. The coloration changed quickly to a dirty green‒brownish tone.

In total, the PEG 200-treated samples were clearly hardened, malformed, and color-changed, and they appeared as if they were fixed in formalin.

### Microscopic examination

**Fig. 9:**
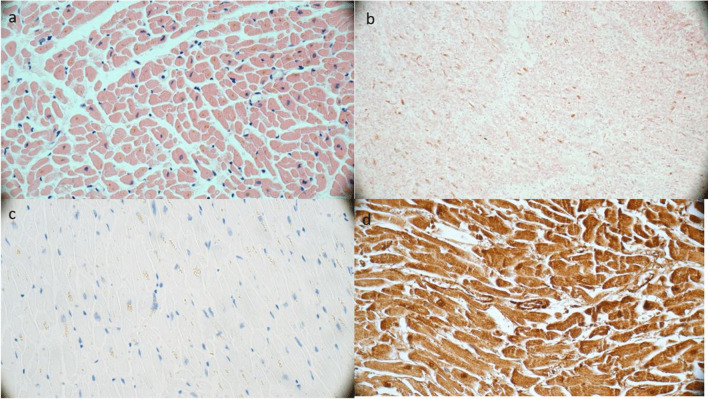
Microscopisc examination of the heart, each 400 × magnification: **a**) heart after 72 h in PEG – **b**) heart after 72 h in oil **c**) heart after 72 h in PEG with anti-C5b9 antibody – **d**) heart after 72 h in PEG with anti-Myoglobin antibody

The microscopic examination of the samples took place after 72 h. First, there were a lot of cutting defects. We then placed the tissue samples in a water bath for several minutes to make more water molecules available for the hygrogropic effect of the PEG. This softened the (solidified) tissue a bit. During the microscopic examination of the samples, all organ, tissue and vessel sections were structurally well assessable. The heart sample still showed the cell cores and the structure of the muscle fibers (Fig. 9a). The vessel walls of the aorta abdominalis and the vena cava inferior were regular, with some atheromatous beds. However, cut artifacts from the microtome were still found, especially in the heart and liver samples. Due to the good quality of the HE-stained sections, we performed immunohistological staining with the following antibodies for the heart (anti-fibronectin, anti-myoglobin (Fig. 9d), anti-C5b9 (Fig. 9c), and anti-CD3) and for the lung (anti-CD3, anti-C5b9, anti-CD15 and anti-NP57). The immunohistological slices were all easily assessable. The sections were positive for the anti-CD15 and anti-NP57 antibodies. Only heart staining with anti-fibronectin and anti-myoglobin showed increased staining of the tissue in the peripheral areas, probably due to autolysis.

All macroscopic documented changes in color and consistency of every tissue are available in Appendix Table 1.

## Discussion

After analyzing and testing different contrast media and different carrier substances at the beginning of the forensic postmortem angiography era, the number of angiographic carrier substances used has decreased to mainly two different substances in recent years. In many forensic institutes, the contrast medium Angiofil® with oil (mostly Paraffinum subliquidum) as a carrier substance is currently used. In contrast, some institutes use a water-soluble, iodine-containing contrast medium, e.g., Accupaque 300®, with polyethyleneglycol 200 as a carrier substance. Some of the different contrast media have been studied and tested in various studies. [3, 4, 8, 14]. Water-soluble iodinated contrast media, e.g., Accupaque 300® with polyethylene glycol 200 as a carrier, have been much less studied than the oily alternative. Mostly radiological artefacts and the behavior of the substance itself in the context of radiological image acquisition and diagnosis were described. [7, 13, 19, 20]. Especially its effects and its influence on the tissues, organs and vessels of the corpse have not been described in detail until now.

It is important to note that postmortem angiography is carried out after external inspection, native CT and subsequent collection of toxicological samples [4, 21]. Most autopsies are performed immediately after PMCTA. For organizational reasons, however, the autopsy may be postponed for a few hours or may not be performed until the next day or the weekend. The contrast medium used in angiography and its carrier substance remain in the body during this time. It is not yet known whether the prolonged time at which the angiographic fluid remains in the body changes the ability to assess the tissues, organs and vessels during autopsy.

The effects of the two relevant carrier substances on the samples that we collected from the corpse were very different.

The organ, tissue and vessel samples of the brain, heart, lung, liver, kidney, spleen and fatty tissue, as well as the samples of the wall of the stomach, the A. carotis, aorta abdominalis and vena cava inferior, which were preserved in oil, showed signs of decomposition after 24 h. The degree of putrefaction even increased after 48 h and 72 h in oil. The signs of putrefaction were detectable both macroscopically and microscopically. However, the samples were stored at room temperature during the observation period. From our own experience, we can assume that the samples would have shown typical decomposition changes after the corresponding periods even without storage in oil [22]. The liquid femoral vein blood formed a heterogeneous mixture of substances with two phases after being added to the carrier substance, which was mixed (temporarily) by shaking the jar. This is an expected process due to the physical and chemical characteristics of the two liquids. Thus, the long-term influence of oil on the tissue appeared to be minimal, and the decomposition changes would most likely have occurred even without the oil. Whether the oil increased the decomposition of the samples could not be determined based on our test set-up. A possible explanation could be the distiribution of bacteria from the vessels of the gut into the general circulation before autopsy, facilitating decomposition. However, this might be prevented with PEG due to its antibacterial properties. However, the unpleasant handling of now slippery and oily tissue surfaces can influence the assessability of organs and different body structures. Our microscopic analysis of the different structures was influenced greatly by decomposition, putrefaction hinders correct histological and immunohistochemical evaluation of tissues, organs and vessels. This has to be kept in mind if the autopsy cannot be performed immediately after the angiography.

Furthermore, it is not possible to assess a potentially fatal fat embolism after pm angiography with oil. Our histological examinations have shown that the diagnosis of fatty liver, necrosis or hepatitis can be difficult or even impossible due to the oil in the vessels.

In contrast, the samples placed in PEG 200 were generally solidified and appeared to be fixed. Polyethyleneglycol has long been used not only in medicine (e.g., as a laxant [23]) but also in preparation, restoration (e.g., in underwater archaeology [24]) and tissue dehydration [25]. Low-molecular-weight PEG, like PEG 200, is a hygroscopic substance that absorbs water (26); thus, tissue can be drained and preserved with this method. The immersion of the different organic samples into the PEG changed both the tactile findings and the visual appearance. These changes can significantly influence the assessability of organs and tissues in the context of an autopsy after angiography with PEG as a carrier substance. It can hinder several diagnoses, especially when thrombi are involved, e.g. fatal lung arterial embolism. Moreover, it is important to keep in mind that the different structures hardened quite quickly by the carrier substance, which can be important for the time setting in organizing a PMCTA with PEG. The microscopic findings appeared to be moderately influenced only by distinct sectional artifacts of individual organs. In contrast, the organ structure itself was not affected. The cutting artifacts during the manufacturing of the microscopic slices could be triggered by the hardened structure of the samples. This could be prevented by rehydration of the tissue in a water bath before the microscopic segments are sliced. The liquid vein blood, which was stored in the PEG 200, showed clear blood agglomerates over time. In particular, the agglutination of PEG with solid blood components such as thrombi makes the assessment of vital thrombi much more difficult. If these agglomerates occur during autopsy due to PMCTA, the assessment is considerably more difficult, especially if the autopsy team is not aware that such artificially generated postmortem clots can be formed by the carrier substance. For example, the diagnosis of a coronary thrombus in cases of sudden cardiac death or after cardiac surgical intervention could be impeded. Further studies could prove that a vital fat embolism can also be reliably diagnosed after a PMCTA with PEG as the carrier substance.

Based on the influence of the vehicle on the assessability of organs and tissues in an autopsy after PMCTA, oil mixed with Angiofil® appears to be preferable for most forensic cases. However, when specific forensic issues concering fat embolism occure, e.g. in specific cases of surgical malpractice or of trauma with question of vital versus post-mortem trauma, or when the liver parenchyma needs to be analyzed in detail (e.g. fatty liver, hepatitis or necrosis), PMCTA with PEG as a carrier is indicated. The use of PEG as a carrier can therefore be a reliable alternative. Other important factors that also significantly influence a PMCTA, such as image quality, cost, storage and handling of substances, were not investigated in this in vitro experiment.

### Limitations

The decomposition of human tissue depends on many factors, including temperature, environment and tissue cohesion. In the present case, a comparative sample was not used due to the many influences on decomposition. However, this means that in our study, we could not investigate the question if the oily liquid leads to a faster decomposition or just does not hinder normal decomposition. In order to answer this question, further studies, comparing the changes of samples with and without exposition to the oil are necessary.

Another limitation is the influence of the contrast agent on the samples, which cannot be estimated in this experiment. When performing a PMCTA with an oil-containing contrast agent mixture, Angiofil® is added to the oil at a dose of 6% [4]. When using PEG, the contrast agent Accupaque® is added to the oil in a mixing ratio of 1:15 (corresponds to 6.67%) [27]. Thus, an influence of the different contrast agents on the organ samples is certainly conceivable, but initially seemed small for our first experiment. However, a further study should investigate the influence of the different contrast agents on the organ samples.

The most important limitation of this study is the fact that it was an in-vitro study with the aim to simply analyze the direct effect of the carrier substances on the tissue. In reality, no one will store the samples inside of the carrier substance, but they are stored in formaldehyde in order to be preserved. This study was not designed to analyses the effect of the carrier substances on the preservation of the samples in a standard formalin fixation.

It is also important to highlight, that it is more realistic to analyze the tissues once the liquid was in the corpse, but it is difficult to perform such a study. Most medico-legal autopsy cases have to be processed as fast as possible. It will not be possible to analyze the tissues of the same body at three different time points as we did.

However, due to our results, more questions arise and justify, in our opinion, further studies to analyze the effect of the different substances in a real-case scenario.

## Conclusion

It is important to choose the most appropriate PMCTA method for each forensic case. Our study provides important information about the influence and effects of the two main carrier substances used to date in forensic medicine on different tissues, organs and vessels.

Indeed, oily contrast mixtures do not modify the macroscopic and microscopic morphology except for the presence of fatty tissue in the organs, which can hinder the diagnosis of fatty embolism and steatosis of the liver. In addition, bodies that underwent PMCTA with oily contrast mixture, are easily concerned by post-mortem changes in the form of putrefaction. A contrast-agent mixture based on PEG, on the other hand, dry and fix the tissues and organs without affecting microscopic accessibility. However, blood agglomerates develop over time and may not be classifiable at autopsy.

Independently of the mixture that is used, we recommend to perform the autopsy and histological sampling as fast as possible after angiography, because, as shown in our study, the contrast agent mixture, independently of its nature, has an influence on the body and the following exams.

This information can help forensic pathologists in their decision-making, coordination and management of their cases. However, further studies are necessary to understand the effect of the different substances in real-case scenarios.

## Appendix

**Table 1:** Overview of the tissue details and their macroscopic changes over time when stored in the carrier substance PEG or Oil for 24 h, 48 h and 72 h

Description	Autopsy*	24 h		48 h		72 h	
		Oil	PEG	Oil	PEG	Oil	PEG
Blood V. cava	Liquid	Liquid oil & blood separates	Small crumbles in liquid blood-PEG mixture	Liquid, oil & blood separates	crumbles in liquid blood-PEG mixture	Liquid, oil & blood separates	crumbles in liquid blood-PEG mixture
Brain	Tough and sticky	reddish discolored, softend tissue	Hardened edges, slight brownish discolored	greenish & reddish discolored, tissue more softened	hardened consistence, brownish discolored	pulpy reddish, hardly identifiable, more softened	hardened consistence, brownish discolored, brighter accentuation of the edges
Heart	no macroscopic infact areas	slight brownish discoloration, no change in consistency	homogeneous brownish discoloration, hardened structure	brown-reddish discolored, softening	homogeneous brownish discoloration, hardened structure	brown-reddish discolored, softened	homogeneous brownish discoloration, hardened structure
Lung	Moderately rich in blood and water	prominent anthracosis, brighter with loss of blood, no change in consistency	homogeneous dark red discolored, rubber-like hardened	sharpened edges of pleura, more softening, dark red discolored	homogeneous dark red discolored, hardened	pleura delimitable, all over dark red homogeneously, softened	homogeneous dark red discolored, hardened
Abdominal Fat	inconspicuous	orange-red discoloration, no change in consistency	brown-orange discoloration, hardened structure	more orange-red discoloration, softening	orange-brown discoloration, hardened structure	homogeneous orange-red discoloration, softened	orange-brown discoloration, hardened structure, shiny aspect
Spleen	Rich in blood, softened	paling, protrusion of the white pulp, no change in consistency	paling, protrusion of the white pulp, easy identifiable, no change in consistency	dark brown, tissue not identifiable, tissue softened	white pulp on bright red structure, hardened,	dark brown, tissue not identifiable, tissue softened	white pulp on red structure, hardened
Liver	No fattening	Homogeneous brown coloration, no change in consistency	bright red-brownish discoloration, hardened edges	Homogeneous dark brown discolored, softened	brownish discolored, hardened structure	Homogeneous darkbrown discolored, softened	brownish discolored, hardened structure
Kidney	Rich in blood	paling, reddish, no change in consistency	homogeneous reddish discolored, hardened edges	overall reddish discolored	homogeneous brownish discolored, hardened structure with prominent edges	overall reddish discolored, softened	homogeneous brownish discolored, hardened structure with prominent edges
Stomach	Folded wall	brownish discolored, no change in consistency	reddish discolored, hardly identifiable, no change in consistency	brownish discolored, hardly identifiable	reddish discolored, hardened, hardly identifiable	brownish discolored, hardly identifiable	reddish discolored, hardened, hardly identifiable
V. cava inferior	Soft wall	tiny gas bubbles under the wall, no change in consistency & color	green-brownish discolored, hardly identifiable, slight hardened structure	tiny gas bubbles under the wall, reddish discolored, no change in consistency	green-brownish discolored, hardly identifiable, hardened structure	reddish discolored, hardy identifiable	green-brownish discolored, hardly identifiable, hardened structure
Aorta abdominalis	Low streaky fat deposits	slightly brown-reddish discolored	brownish discolored, hardened, crumbled, highlighted fat deposits	overall brown-reddish discolored, no change in consistency	brownish discolored, hardened overall, crumbled, highlighted fat deposits	overall brown-reddish discolored, softened	brownish discolored, hardened overall, crumbled, highlighted fat deposits
A. carotis	Low streaky fat deposits	slightly reddish on the edges, no change in consistency	hardened overall, crumbled, brownish discolored	overall reddish discolored, no change in consistency	hardened overall, crumbled, brownish discolored	overall reddish discolored, wavy structure	hardened overall, crumbled, brownish discolored

*for all the blood, tissue and organ sample, only the abnormalities in the autopsy regarding color and haptic are described here. If no description is given, it can be assumed that the sample corresponded to a healthy reference sample
